# Intraosseous Myofibroma of the Mandible in a Pediatric Patient: Comprehensive Cone Beam Computed Tomography (CBCT) and Immunohistochemical Characterization

**DOI:** 10.7759/cureus.112893

**Published:** 2026-07-17

**Authors:** Maria Alphonsa Deepthi, Shyam NDVN, Kiran Kumar Gundamaraju, Vaishali Narayen, Paremala K

**Affiliations:** 1 Department of Oral and Maxillofacial Pathology, Government Dental College and Hospital, Hyderabad, IND

**Keywords:** cbct, intraosseous, mandible, myofibroma, pediatric

## Abstract

Intraosseous myofibroma of the mandible is a benign myofibroblastic neoplasm with limited documentation in the pediatric population. This case report outlines the presentation of a nine-year-old girl with a history of swelling and pain involving the lower left side of the face for two months. Examination revealed a mass originating from the posterior part of the mandible, and radiographic evaluation with panoramic radiography and cone beam computed tomography (CBCT) revealed a well-defined unilocular radiolucency with three-dimensional cortical expansion. Pathological examination revealed the hallmark biphasic pattern of myofibroma, characterized by peripheral zones of spindle-shaped myofibroblastic cells arranged in short fascicles and central areas of immature round to oval cells with a hemangiopericytoma-like vascular pattern. Immunohistochemical analysis demonstrated diffuse strong positivity for vimentin and α-SMA, weak positivity for desmin, and negative for S-100 protein, confirming myofibroblastic lineage (the vimentin-actin-desmin cytoskeletal phenotype of myofibroblast) while systematically excluding nerve sheath tumors such as neurofibroma and muscle tumors such as leiomyoma. Conservative surgical excision was performed with preservation of adjacent developing dentition, and the patient is under active follow-up. This case underscores the critical role of histopathological and immunohistochemical evaluation in the definitive diagnosis of intraosseous myofibroma, particularly in differentiating it from a broad spectrum of morphologically similar intraosseous lesions in the growing mandible.

## Introduction

The term "myofibroma" was introduced by Smith et al. in 1989 to describe a rare benign spindle cell neoplasm of myofibroblastic origin [1]. Myofibroblasts exhibit a dual phenotype combining fibroblastic and smooth muscle cell features [2]. A myofibroma predominantly affects the head and neck region, followed by the trunk and extremities [3].

According to the World Health Organization (WHO) Classification of Head and Neck Tumors, 5^th^ edition (2022), a myofibroma is classified as a benign pericytic (perivascular) tumor based on its shared morphologic and molecular features with myopericytoma [4]. Owing to its infrequent occurrence coupled with overlapping clinical and radiographic features with other odontogenic, benign mesenchymal, and low-grade malignant tumors such as dentigerous cyst, ameloblastoma, desmoplastic fibroma, and fibrosarcoma, a myofibroma carries a significant risk of misdiagnosis, making histopathological and immunohistochemical evaluation indispensable for definitive diagnosis [5].

A comprehensive compilation of 46 reported pediatric intraosseous myofibroma cases has been documented by Lazim et al. (2024) [6]. Subsequent to this, a few additional cases have been reported.

The present case constitutes an additional contribution to this series by its three-dimensional cone beam computed tomography (CBCT) analysis, expanded immunohistochemical characterization, and postoperative follow-up radiographic assessment demonstrating satisfactory healing without recurrence.

## Case presentation

A nine-year-old female patient with no relevant medical, dental, or family history presented with a history of swelling and pain in the lower side of the face for two months. The swelling was gradual in onset and was smaller in size initially and gradually progressed to the present size of 4.0 x 4.0 cm. The swelling was associated with pain, which subsided with medication from the medical hospital. A history of similar swellings was ruled out elsewhere in the body.

Extraoral examination showed gross facial asymmetry on the left side of the face. A diffuse solitary swelling of approximately 4.0 x 4.0 cm was noticed in the lower left third of the face, extending anteroposteriorly from the left corner of the mouth to 2 cm anterior to the ramus. Superoinferiorly from the inferior border of the mandible to the left zygoma. Overlying skin was normal with no secondary changes. On palpation, the swelling was non-compressible, non-fluctuant, and non-pulsatile. Temporomandibular joint movements were normal. Left submandibular lymph nodes were palpable (Figure 1A).

**Figure 1:**
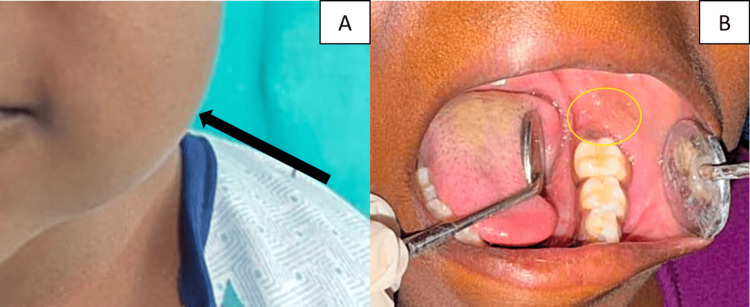
A) Clinical image showing a solitary swelling in the lower left third part of the face. B) Clinical image showing a mass on the left posterior region in relation to the permanent mandibular first molar extending to the ascending ramus (marked by a yellow circle).

On intraoral examination, mouth opening was adequate, and swelling was noticed in relation to the left posterior region extending from the permanent mandibular first molar to the ascending ramus. On palpation, swelling was non-tender, non-compressible, non-fluctuant, and firm to hard in consistency. Mucosa over the swelling was normal with vestibular obliteration in relation to the permanent mandibular left first molar region and tender on percussion (Figure 1B).

On radiographic examination, a well-defined radiolucency was seen extending anteroposteriorly from the mesial aspect of the mandibular first molar (36) till 1 cm in front of the posterior border of the ramus of the mandible and superoinferiorly 0.5 cm away from the alveolar crest till 0.6 cm away from the lower border of the mandible, measuring about 3.5 x 2.4 cm. It also showed thinning and loss of both buccal and lingual cortical plates, with perforation seen along the lower border of the mandible. The radiograph also denoted that the patient was in the mixed dentition stage appropriate for her chronological age, with a partially formed mandibular second molar crown at the superior aspect of the lesion and open apices seen in relation to the mesial and distal roots of the mandibular left first molar with no evidence of delayed eruption, missing teeth, or malpositioning of teeth (Figures 2-4).

**Figure 2:**
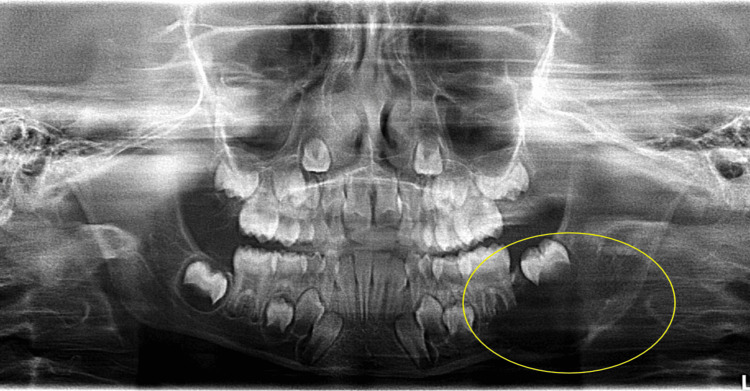
X-ray of the mandible showing a well-defined radiolucency extending anteriorly from the mesial root of the permanent mandibular left first molar to the angle of the mandible distally, with a partially formed mandibular second molar crown at the superior aspect of the lesion and open apices seen in relation to the mesial and distal roots of the mandibular left first molar (marked by the yellow circle).

**Figure 3:**
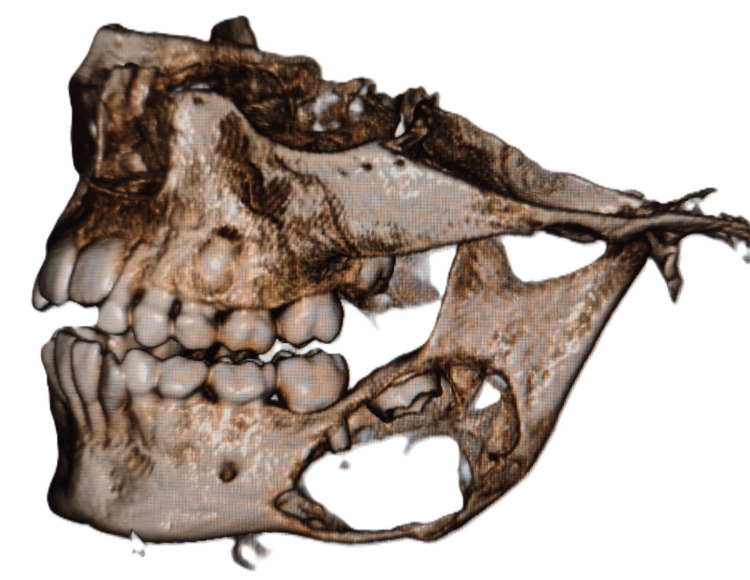
Radiograph of the mandible showing a well-defined radiolucency extending anteriorly from the periapical region of the permanent mandibular left first molar to approximately 2 cm posterior to the sigmoid notch.

**Figure 4:**
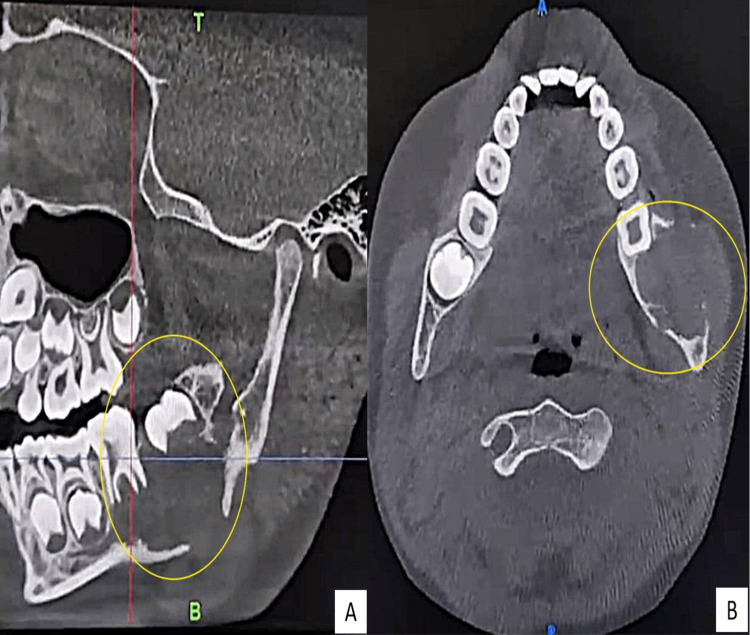
X-ray of the mandible showing a well-defined expansile osteolytic lesion. A) The lesion extends anteroposteriorly from the mesial aspect of the mandibular first molar (36) to approximately 1 cm anterior to the posterior border of the mandibular ramus, and superoinferiorly from 0.5 cm below the alveolar crest to 0.6 cm above the inferior border of the mandible, measuring approximately 3.5 × 2.4 cm (yellow circle). (B) Buccolingual expansion with thinning of the buccal and lingual cortical plates (yellow circle).

On the basis of the above clinical and radiographic findings, a provisional diagnosis of ameloblastoma in relation to the posterior mandible was made. An incisional biopsy from the site of the left angle of the mandible was done.

On gross examination, multiple creamish brown soft tissue bits measuring about 12.5 x 3.3 cm, which were firm in consistency, were received (Figure 5).

**Figure 5:**
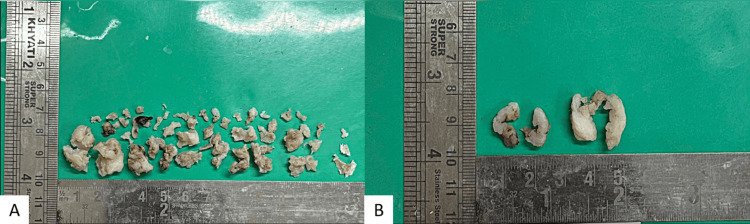
A) Multiple creamish brown soft tissue bits (37 soft tissue bits) and hard tissue bits (three bone bits) altogether measuring about 12.5 x 3.3 cm; the soft tissue bits were firm in consistency. B) Two soft tissue bits were cut into four pieces.

Microscopic evaluation of soft tissue sections demonstrated connective tissue stroma comprising plump, spindle-shaped cells arranged in the form of streaming fascicles and whorls. The cells appeared as oval to spindle-shaped plump fibroblast-like cells with a variable amount of collagen and vasculature. The cells presented a characteristic biphasic arrangement with a peripheral zone composed of spindle-shaped cells and a central zone composed of more primitive-looking round to oval cells with few capillaries showing irregular branching (hemangiopericytoma-like vasculature). There was evidence of mild cellular and nuclear atypia in the form of pleomorphism, hyperchromatism, and prominent nucleoli. A focal area of calcification was seen. The intervening stroma comprised a moderate amount of chronic inflammatory cell infiltrate. Areas of myxoid and primitive ectomesenchymal tissues were evident. Despite focal mild nuclear pleomorphism, the absence of mitoses was consistent with a benign tumor (Figure 6).

**Figure 6:**
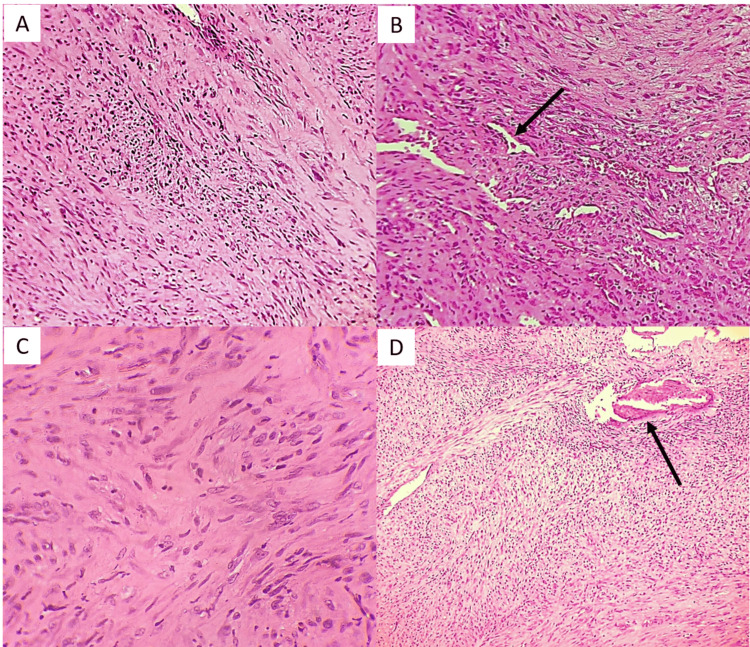
A) Photomicrograph showing alternate areas of polygonal cells and spindle cells with elongated nuclei (H&E, 20X). B) Photomicrograph showing capillaries with an antler-like pattern (arrow points to antler-like capillary) (H&E, 20X). C) Photomicrograph showing plump spindle-shaped cells with focal mild pleomorphism, hyperchromatic nucleus, and prominent nucleoli (H&E, 40x). D) Photomicrograph showing calcification (arrow pointing to calcification) (H&E, 20X).

Immunohistochemical analysis was performed to determine the lineage of spindle cells. The spindle cells demonstrated positivity for vimentin, α-SMA, patchy weak positivity for desmin, and negativity for S-100 (Figure 7). The absence of neural tissue marker expression and weak patchy positivity of desmin excluded nerve tumors such as neurofibroma and schwannoma and muscle tumors such as leiomyoma, respectively. Based on the cumulative histopathological and immunohistochemical findings, a final diagnosis of intraosseous myofibroma was established.

**Figure 7:**
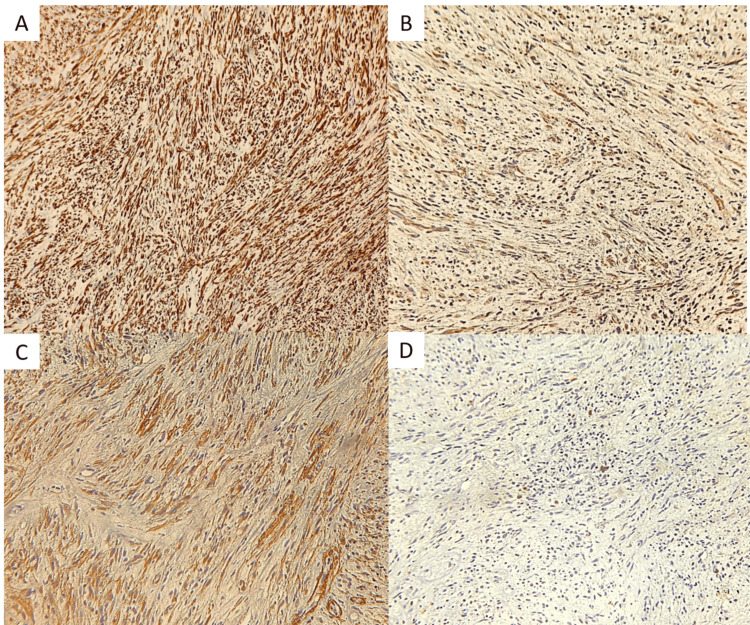
A) Immunohistochemistry showing spindle cells with strong positivity for vimentin (20X). B) Immunohistochemistry showing spindle cells with weak positivity for desmin (20X). C) Immunohistochemistry showing spindle cells with positivity for alpha-smooth muscle actin (20X). D) Immunohistochemistry showing spindle cells with negativity for S-100 (20X).

Conservative surgical excision was performed with preservation of adjacent developing dentition. The patient is under active follow-up.

A follow-up panoramic radiograph obtained one month postoperatively revealed the surgical site to be in the process of healing with a faint trabecular pattern near the margins of the lesion noted at the defect site. Complete bony fill was not yet evident; however, the healing was progressing satisfactorily with no signs of residual or recurrent lesion. The inferior alveolar nerve canal is traceable, coursing below the developing mandibular molar crown (Figure 8).

**Figure 8:**
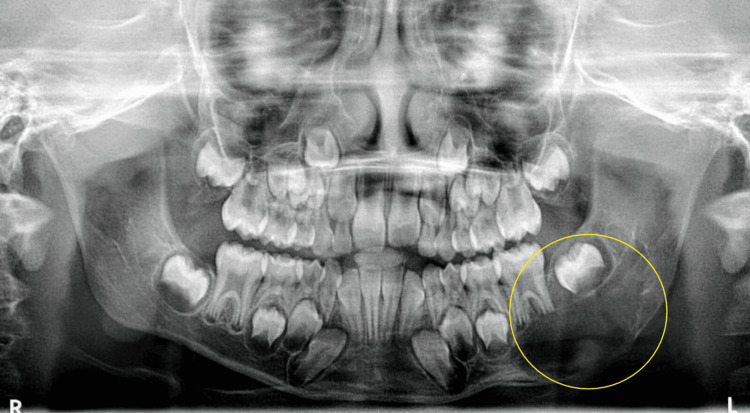
Postoperative radiograph of the mandible showing a faint trabecular pattern near the margins of the lesion is suggestive of the initiation of osseous healing, with the inferior alveolar nerve canal being traceable (marked by a yellow circle).

## Discussion

A myofibroma is a benign mesenchymal neoplasm of myofibroblastic origin, occurring either as a solitary lesion (myofibroma) or as multicentric lesions (myofibromatosis). The historical evolution of its nomenclature reflects the changing understanding of its biological nature. In 1951, Jo W and Schrum initially described the lesion as congenital fibrosarcoma owing to its spindle cell morphology and presumed malignant behavior [7]. Subsequently, Stout reclassified it in 1954 as congenital generalized fibromatosis, recognizing its benign yet multicentric growth pattern [8]. Later, Chung and Enzinger introduced the term "infantile myofibromatosis" in 1981 after demonstrating its characteristic myofibroblastic differentiation [9]. Then the term "myofibroma" was introduced by Smith et al. in 1989 [1].

Myofibromas commonly occur in the head and neck region, trunk, and extremities. Although soft tissues represent the most frequent site of occurrence, intraosseous presentation and involvement of the mandible are uncommon. Myofibromas may also develop in visceral locations, including the musculature, central nervous system, pancreas, lungs, and heart [10].

Myofibromas of the mandible are tumors typically observed in children, with a mean age of 7.2 years. It commonly occurs in the first decade of life, showing a clear male predominance with a male-to-female ratio of 2.3:1 [11]. A comprehensive compilation of 46 reported pediatric intraosseous myofibroma cases has been documented by Lazim et al. [6]. Subsequently, three cases and one case series have been reported in the literature, excluding our case (Table 1) [12-14].

**Table 1:** Demographic, clinicoradiographic, and treatment characteristics of three pediatric patients with mandibular myofibroma.

Author	Patient age/sex	Size	Radiographic investigations	Treatment
de Macêdo Santos et al., 2024 [12]	Two-year-old/Male	3.2x2.9x2.5 cm	Well-defined intraosseous contrast-enhancing mass along the right mandibular ramus	Incisional biopsy followed by total enucleation
Loh et al., 2025 [13]	15-year-old/Male	5.0x4.0x6.0 cm	Well-defined lucent lesion within the mandibular body	Marginal mandibulectomy
Alpaguter et al., 2025 [14]	Six-year-old/Female	Fixed palpable mass of height 0.2-0.3 cm	Radiolucent lesion with impacted premolars along with lingual plate perforation	Wide surgical excision.

Further supporting the clinicopathological profile of this entity, Barajas-Torres et al. recently published the largest institutional series of jaw myofibromas in pediatric patients to date, comprising 14 cases with a median age of 6.5 years, confirming mandibular predominance and consistent SMA positivity across cases [15].

The molecular basis of myofibroma remains incompletely understood. Germline PDGFRB mutations have been identified in rare familial cases of infantile myofibromatosis, whereas most infantile and adult solitary myofibromas are sporadic, arising from somatic PDGFRB mutations. A subset of cellular myofibroblastic tumors has additionally been shown to harbor somatic serum response factor (SRF) gene rearrangements, most commonly SRF-RELA fusions. SRF is a transcription factor that regulates smooth muscle differentiation, and tumors carrying these fusions display a more myoid immunophenotype with variable desmin expression. This is consistent with the vimentin-actin-desmin cytoskeletal profile of myofibroblasts [16].

Intraosseous myofibromas most commonly appear on radiographic examination as circumscribed, unilocular radiolucent lesions [11]. Nevertheless, variations in presentation have been reported, including multilocular morphology, displacement of adjacent teeth, expansion of cortical plates, and focal cortical breach [11, 17]. In the present case, the lesion demonstrated radiographic features largely consistent with previously reported findings, presenting as a unilocular radiolucency that had enlarged considerably and extended toward the angle of the mandible, with thinning of the buccal and lingual cortical plates and perforation of the lower border of the mandible but without any displacement of the adjacent teeth.

Notably, dentition-related changes have been observed in cases occurring during the mixed dentition period, with Lazim et al. reporting malposition of the unerupted canine and premolar in a nine-year-old patient [6], and Cunha et al. reporting displacement of the canine and both premolars in a nine-year-old patient [18]. In contrast, the present case demonstrated a normal eruption pattern with no evidence of malpositioning or missing teeth, despite the considerable size and posterior extent of the lesion, suggesting that displacement of teeth and eruption disturbance, while occasionally reported, are not invariable features of intraosseous myofibroma even in extensive lesions.

Histopathologically, a myofibroma characteristically demonstrates a biphasic architectural pattern composed of two distinct cellular areas. The peripheral region is generally less cellular and is formed by interlacing fascicles of spindle-shaped cells exhibiting elongated vesicular nuclei, poorly defined cell margins, and eosinophilic cytoplasm [18]. In comparison, the central component tends to be more cellular, consisting predominantly of round to spindle-shaped cells with hyperchromatic nuclei and minimal cytoplasm, often arranged around prominent thin-walled branching hemangiopericytoma-like vascular channels [10].

Immunohistochemical evaluation serves as an essential adjunct in differentiating myofibroma from other benign and malignant spindle cell neoplasms. Tumor cells in myofibroma commonly demonstrate immunoreactivity for α-SMA and vimentin, supporting their myofibroblastic differentiation. In the present case, a weak positivity for desmin demonstrates the vimentin-actin-desmin cytoskeletal phenotype of myofibroblasts. The absence of strong diffuse desmin positivity effectively excluded leiomyoma, in which desmin is characteristically expressed strongly, and negative immunostaining for S-100 further aided in excluding lesions of neural origin such as neurofibroma and schwannoma [19].

The histopathological differential diagnosis of spindle cell lesions, together with the immunohistochemical marker panel employed to determine the nature of the neoplastic cells, has been listed here (Table 2) [10].

**Table 2:** Histopathological differential diagnosis of a myofibroma based on morphological and immunohistochemical features.

Pathology	Microscopic distinguishing features	Immunohistochemistry
Leiomyoma [10]	Spindle cells with blunt-ended cigar-shaped nuclei with perinuclear vacuoles arranged as well-defined intersecting fascicles and no biphasic pattern.	Positive for α-SMA, desmin, h-caldesmon, MSA, calponin
Fibromatosis [10]	Slender spindle cells arranged in long sweeping fascicles separated by abundant collagen showing infiltrative growth and no biphasic pattern.	Positive for vimentin, beta-catenin
Fibrous histiocytoma [10]	Spindle cells arranged in a storiform pattern. Histiocytes, xanthoma cells, siderophages, and Touton giant cells may be seen.	Positive for vimentin, CD-68
Nodular fasciitis [10]	Prominent myxoid matrix, tissue culture-like arrangement of spindle cells intermixed with lymphocytes and erythrocytes, no hemangiopericytoma-like vasculature.	Positive for vimentin, α-SMA, MSA, and USP6 rearrangement
Solitary fibrous tumor [10]	Patternless architecture of spindle cells, staghorn vessels, no biphasic pattern.	Positive for CD34, STAT6 nuclear
Schwannoma [10]	Alternating Antoni A and B areas, Verocay bodies formed by two rows of palisading nuclei are seen and encapsulated.	Positive for S100 strongly, SOX 10. Negative for α-SMA
Neurofibroma [10]	Wavy dark stained nuclei, wire-like collagen strands resembling shredded carrots, and mast cells are seen and not encapsulated.	Positive for S100 (variable), SOX 10. Negative for α-SMA
Low-grade myofibroblastic tumor [10]	Nuclear atypia, infiltrative margins into surrounding soft tissue, and tram-track actin staining are seen; no biphasic pattern.	Positive for vimentin, α-SMA (tram track pattern), desmin (<50%), calponin. Negative for ALK
Inflammatory myofibroblastic tumor [10]	Prominent lymphoplasmacytic infiltrate, spindle cells arranged in a storiform or fascicular pattern, histiocytoid cells may be seen.	Positive for α-SMA, MSA, desmin, ALK (36-60%)

Conservative surgical excision remains the treatment of choice for solitary myofibromas. However, in extensive lesions associated with significant tissue destruction, more aggressive surgical approaches, such as segmental resection of the jaw, may be necessary. As mandibular myofibromas frequently occur in pediatric patients, reconstructive procedures are often required to restore both function and anatomical integrity. Tumor recurrence following surgical excision is uncommon and is generally associated with anatomically complex cases in which complete removal of the lesion is challenging [20].

The present case bears notable similarity to a recently reported case by Lazim et al., who described intraosseous myofibroma of the mandible in a nine-year-old female patient, further highlighting the occurrence of this entity in the pediatric population and its predilection for the mandible [6]. Building upon the findings of Lazim et al., the present case contributes several distinguishing clinicopathological observations that warrant consideration. While the aforementioned case involved the parasymphysis region of the body of the mandible, the present case arose in the body, angle, and ramus region, illustrating that intraosseous myofibroma, despite its overall predilection for the mandible [11], can present at anatomically distinct subsites with differing surgical implications. The posterior subsite in the present case carries greater surgical complexity owing to its close proximity to the inferior alveolar nerve, the masseteric musculature, and the developing dentition. From a diagnostic standpoint, the present case expands upon the immunohistochemical panel of the previously reported case, which was limited to α-SMA and Ki-67. Further characterization was achieved through inclusion of vimentin, desmin, and S-100 protein, enabling a more systematic exclusion of histological mimics. Vimentin confirmed the mesenchymal lineage, weak desmin positivity reflected the vimentin-actin-desmin cytoskeletal phenotype, and S-100 negativity effectively excluded nerve sheath tumors, an important consideration given the proximity of the lesion to the inferior alveolar nerve. CBCT analysis provided precise delineation of buccolingual cortical involvement and true lesion extent. Furthermore, the post-operative panoramic radiograph demonstrated a faint trabecular pattern near the peripheral margins of the surgical site, suggesting initiation of osseous healing. Collectively, these findings build upon previously reported cases and contribute to the evolving clinicopathological characterization of intraosseous myofibroma of the pediatric mandible.

## Conclusions

An intraosseous myofibroma should be recognized as an important diagnostic consideration in the evaluation of radiolucent jaw lesions in pediatric patients. This case emphasizes that a definitive diagnosis cannot be established solely on clinical or radiographic findings; instead, histopathological examination complemented by immunohistochemical analysis remains essential. The characteristic expression of α-SMA supports myofibroblastic differentiation and aids in distinguishing myofibroma from a broad spectrum of morphologically similar spindle cell neoplasms. Increased awareness of this rare entity among oral pathologists, oral and maxillofacial surgeons, and pediatric dentists is crucial to facilitate accurate diagnosis, guide appropriate conservative management, and avoid unnecessary aggressive surgical interventions.
